# Quit tobacco clinics in Bahrain: smoking cessation rates and patient satisfaction

**DOI:** 10.1186/s12971-017-0115-1

**Published:** 2017-01-21

**Authors:** Randah Ribhi Hamadeh, Jamil Ahmed, Maha Al-Kawari, Sharifa Bucheeri

**Affiliations:** 1 Department of Family and Community Medicine, College of Medicine and Medical Sciences, Arabian Gulf University, P.O.Box 26671, Manama, Bahrain; 2 Quit Tobacco Clinics, Ministry of Health, P.O.Box: 11464, Manama, Kingdom of Bahrain

**Keywords:** Tobacco, Cigarettes, *Shisha*, Waterpipe, Cessation, Quit smoking

## Abstract

**Background:**

One third of Bahraini adult males and 7.0% of females use all types of tobacco. The prevalence rates of cigarette and *shisha* smoking are 11.0 and 6.0%, respectively. Tobacco cessation programs are essential to help smokers quit. The objectives of this study were to determine the quit rates among male attendees of quit tobacco clinics (QTC) in Bahrain and describe related factors.

**Methods:**

We used a cross sectional study design to interview194 male tobacco smokers who had received care from two QTC. Patients who consulted these clinics within the year preceding the study were eligible to be included. They were interviewed using a structured and pretested questionnaire containing questions on tobacco smoking behavior and quitting experience.

**Results:**

Overall, 56.5% had quit all forms of tobacco after attending the QTC with s*hisha* smokers being more successful in quitting than cigarette smokers. About 93.0% received nicotine replacement treatment along with counseling sessions. More than three visits to the clinics and previous quit attempts of 21 months duration or more were statistically significantly related to successfully quitting all types of tobacco (*p* < 0.05). Most participants were satisfied with the clinics; however the majority wanted longer opening hours and an increase in the working days of the clinic. Physicians referred only 18.0% of the study population to QTC.

**Conclusion:**

A high tobacco-quit rate among smokers seeking treatment at QTC is encouraging and indicates that the clinics contributed to tobacco cessation in Bahrain. Counselling sessions and more frequent visits to QTC helped participants to successfully quit tobacco.

## Background

Over one third (35.8%) of males and 6.6% of females smoke tobacco globally with corresponding proportions of 36.8 and 2.8% in the Eastern Mediterranean Region (EMR) [1]. The successful smoking cessation or tobacco dependence treatment can restore health status back to normal to a great extent. The risk of premature death and morbidity is reduced by 90.0% if smokers quit before the age of 30 or by 50.0% if they quit by the age of 50 [2]. Appropriate smoking cessation services including national quit line, nicotine replacement therapy (NRT) and other cessation cost-covered services are now available, mostly in developed countries. But the progress towards tobacco cessation has been very slow since 2010 [1]. In the six Gulf Cooperation Council (GCC) countries tobacco cessation services are available since 2014 [3].

The tobacco smoking rates are still high in most EMR countries; including GCC countries, despite serious smoking control efforts [4]. The tobacco prevalence in the region is about 32.0% for adults (≥15 years) and 36.0% for youth between 13 to15 years and it is on the increase in younger populations. Notably, the age standardized prevalence smoking rates among males are very high in Jordan (43.0%) and Tunisia (45.0%) [2]. Waterpipe smoking in particular, is increasing in youth of most countries of the region as 39.0% and 31.0% of boys and girls, respectively, smoke it [5].

Quitting tobacco is often challenging for smokers without support [4, 6]. However, about 84.0% of smokers can quit if they receive advice from a physician [1]. In 2013, the age standardized prevalence of current use of any tobacco form was 30.3, 7.1 and 42.7% among adults of sexes combined, males and females, respectively in Bahrain [1]. Significant proportions of adult male (11.0%) and female Bahraini (6.0%) are current *shisha* smokers [7]. Lung cancer is the most common cancer in Bahraini males and second in females with annual age standardized incidence rates of 31.1/100,000 and 10.7/100,000, respectively [8]. It has also been reported that more than 200 deaths are attributable to tobacco related diseases in Bahrain annually [9].

Dedicated tobacco cessation clinics are necessary to help tobacco users quit. Bahrain ratified the World Health Organization’s Framework Convention on Tobacco Control in 2007 and passed an antismoking law in 1994 with a modified one in 2009 [4]. Tobacco advertisement, promotion and sponsorship are banned in the country [10]. QTC were established in three health centers in the Kingdom since 2004 to provide services to residents wishing to quit smoking. The first clinic, Hoora was established in 2004 followed by Hamad Kanoo (2012) and Bank of Bahrain and Kuwait (2014). Although the services including consultation and NRT are given free of cost, the mean consultation cost, incurred by the Ministry of Health (MOH), for advice on smoking cessation is 4.5 Bahraini Dinar (BHD) (12 USD) excluding that of NRT. No efforts were made prior to our study to assess the usefulness of these clinics in smoking cessation. Such studies are essential for health policy makers in their tobacco control efforts; not only in Bahrain but also in other GCC countries. The objectives of the study were to determine the smoking quit rates among males attending the QTC, assess their satisfaction with the treatment services, determine their smoking behavior prior to consultation and describe the sociodemographic determinants of smoking cessation.

## Methods

**Fig. 1:**
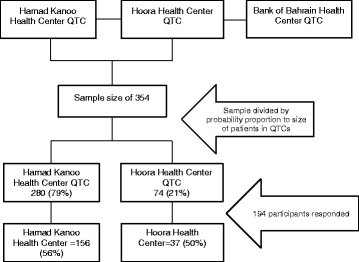
Study population and sampling schematic

This cross sectional study was conducted between 10 August and 30 December 2015. A sample of 354 male patients was estimated and stratified based on the proportion probability to size in the two QTC; Hoora (280) and Hamad Kanoo (74) health centers. All males who attended these clinics in 2013 and 2014 were eligible for inclusion (Fig. 1). As females made a negligible proportion of the clinic attendees, they were excluded; so was the QTC at the Bank of Bahrain and Kuwait health center as it was recently established, in 2014.

The data were collected through a questionnaire, in Arabic or English, and both versions were pilot tested on 10.0% of the clinic attendees who were excluded from the study. Patients were selected through simple random sampling method from the clinics’ registers. The participants were called by phone and data collectors requested them to visit the clinics for interview after informing them about the objectives of the study and that participation was voluntary. The questionnaire had no identity verification and strict confidentiality of the participants was ensured. Interviews were performed by the trained QTC auxiliary staff. Consent forms were signed by the study participants before the interviews.

A smoker was considered a quitter if he stopped smoking any tobacco product for at least six months after attending the clinic. Relapse in tobacco smoking was defined as resuming smoking after a complete abstention for at least a month. Descriptive analysis was done to present frequencies of sociodemographic, smoking behavior and quit tobacco related variables. Chi square or *t*-test was used to draw inferences about categorical and continuous variables, respectively.

## Results

Data were available for 194 cases from the original sample. Overall, the response rate was 54.8%, with a slightly higher percentage from Hoora Clinic (56.1%) than Hamad Kanoo (50.0%) (Fig. 1). The majority of the study participants were Bahraini (80.6%), and the rest were other Arab and South Asians. Their mean age was 37.2 years with 37.3% having secondary education or higher and almost one third semi-skilled jobs and 30.4% had ever been unemployed (Table 1). The average monthly income of the 95 who disclosed it was 781.1 ± 535 BHD (2071.91 ± 1419.12 USD).

**Table 1:** Sociodemographic characteristics of the patients attending the QTC

Characteristic	Mean	±1SD
Age of participant (*n* = 191)	37.2	13.9
Total years of education (*n* = 190)	12.7	3.3
Number of children (*n* = 132)	3.5	1.7
Income (BHD) per month (*n* = 95)	781.1	535.1
Number of clinic visits	2.7	2.0
Previous unemployment (years) (*n* = 55)	2.0	3.0
Nationality (*n* = 175)	n	%
Bahraini	141	80.6
Other Arab	21	12.0
Others	13	7.4
Educational level (*n* = 193)		
Primary and below	17	8.8
Intermediate	22	11.4
Secondary	82	42.5
Graduate and above	72	37.3
Occupation (*n* = 194)		
Low professional	20	10.3
Skilled	31	16.0
Semi-skilled	56	28.9
Unskilled	8	4.1
Unemployed	4	2.0
Retired	25	12.9
Student	31	16.0
Unspecified	19	9.8
Ever unemployed (*n* = 194)		
Yes	59	30.4
No	135	69.6

### Sources of information about the QTC and referrals

The participants’ main source of information of the QTC was their friends (36.1%) followed by the primary healthcare establishment (25.1%), physician (7.3%) or other (17.8%). The majority (96.9%) of the patients were referred to QTC by their friends, wives and physicians. Further, 25 were referred due to illness, mainly diabetes mellitus (40.0%) and lung diseases (28.0%).

### Quit rates and reasons for quitting

**Fig. 2:**
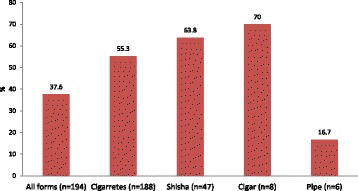
Tobacco quit rates for six months or more at the Quit Tobacco Clinics by type of tobacco smoking (*n* = 194)

On an average, the participants made 5.2 ± 11.0 and 3.0 ± 2.2 attempts to quit cigarettes (*n* = 185) and *shisha* (*n* = 41) smoking, respectively. Overall, 56.5% quit all forms of smoking during the study period with 37.6% quitting for six months or longer, after seeking the QTC services. The highest quit rate was among cigar smokers (70.0%) followed by *shisha* (63.8%), cigarette (55.1%) and pipe (16.7%) smokers (Fig. 2).

Health (76.0%) and family (15.7%) were the main reasons, for the participants to quit tobacco smoking. Family (57.6%) and physicians (33.0%) were the main support for those who quit tobacco smoking. Personal problems (34.2%), habit (27%), friends (11.7%), enjoyment (11.7%) and nervousness (8.1%) were identified as the main reasons for not being able to quit more than one form of tobacco.

### Relapse and withdrawal

Sixty-three participants (35.4%) experienced relapse and started smoking again after successfully quitting. Personal problems (55.0%), friends (15.0%), loneliness (11.3%) and withdrawal symptoms (9.4%) were the reasons for the failure to sustain successful quit attempts. Headache (40.3%) and nervousness (42.9%) were the commonest withdrawal symptoms experienced by the quit attempters.

### NRT

One hundred eighty-one participants received NRT, mostly nicotine chewing gums and patches combined (79%), with only 3.6% given either Bupropion or Champix. One person used traditional herbal treatment to quit tobacco. Only 11.7% purchased NRT from a private pharmacy while the remaining received it for free from the QTC. For those who purchased NRT out of pocket, the cost was unaffordable to 7.2%. When asked if they had to buy NRT, if not provided by the clinic, 51.5% said that they would buy it.

### Participants’ smoking behavior prior to consultation

There were 188 cigarette smokers (96.9%) and 6 did not smoke cigarettes at all. 74.5% of the cigarette smokers smoked cigarettes only and the rest smoked it in addition other tobacco products. Forty-seven smoked *shisha* (23.2%), of whom 5 (10.5%) smoked *shisha* only and 37 (79.0%) smoked it along with cigarettes and 5 (10.5%) along with cigarettes and other types. Only one smoker smoked other tobacco products only (cigar) while the rest (cigar and pipe smokers) smoked cigarettes or shisha along with that product. The age of starting any type of tobacco smoking was 16.4 ± 7.8 years.

### Cigarette and *shisha* smokers

Participants in our study started smoking cigarettes at an average age of 15.9 ± 4.4 years, smoked 27 ± 16.9 cigarettes per day, for 19.8 ± 12.8 years and spent 1.2 ± 0.7 BHD (3.2 ± 1.9 USD) daily on cigarettes. However, the mean age of starting to smoke, duration of smoking and cost were statistically significantly different when comparing cigarette and *shisha* smokers (*p* ≤0.001). The most common reason to start smoking cigarettes was friends (46.8%) or experimentation (29.3%) with LM (33.0%) and Marlboro (32.0%), the commonest brands smoked. Similarly friends (61.0%) and experimentation (13%) were the main reason to start *shisha* smoking. *Fakher* and *Nakhla* tobacco brands were smoked by 55% and 45%, respectively with grape (50.0%), apple (29.5%) and pomegranate (13.6%) the most smoked *shisha* flavors. Cigarette smokers had stronger craving reflecting very high tobacco dependence than the *shisha* smokers (*p* < 0.001) (Table 2).

**Table 2:** Comparison of cigarette and *shisha* smoking behavior

	Cigarettes (*n* = 188) Mean ± 1SD	*Shisha* (*n* = 47) Mean ± 1SD
Age started smoking in years (*n* = 185)	15.9 ± 4.4	19.7 ± 7.4
Number smoked (*n* = 188)*	27 ± 16.9 (day)	3.4 ± 4.5 (week)
Duration of smoking in years (*n* = 177)*	19.8 ± 12.8	8.2 ± 6.1
Smoking cost (BHD) (*n* = 187)*	1.2 ± 0.7 (day)	3.1 ± 3.3 (weekly)
	*n* (%)	*n* (%)
Time of first cigarette/*shisha* smoked on a typical day*		
Within 30 min of waking up	145 (77.6)	1 (2.1)
> 30 min of waking up	42 (22.4)	46 (97.9)
Smoking in morning more than rest of the day*		
Yes	65 (34.9)	1 (2.1)
No	121 (65.1)	46 (97.9)
Cigarette/*shisha* most hate to give up*		
First in the morning	73 (38.6)	0 (0.0)
Any other	116 (61.4)	42 (100)
Smoked cigarettes during illness*		
No	94 (50.8)	43 (91.5)
Yes	91 (49.2)	4 (8.5)
Difficulty avoiding smoking in prohibited areas*		
No	116 (61.4)	45 (95.7)
Yes	73 (38.6)	2 (4.3)

*P < 0.001

Mean age, total years of education, income and number of children were not statistically significantly different among those who had quit all forms of tobacco compared to those who did not. Similarly, the mean age at starting cigarette smoking, number of cigarettes smoked per day, cigarette smoking quit attempts, duration and cost of cigarettes per week, total years of *shisha* smoking, number of *shisha* smoked per week, *shisha* quit attempts and cost of *shisha* were not statistically significant among the two groups.

We found that those who quit all forms of tobacco were more likely to have been unemployed, smoked cigarettes even when they were sick, were unable to quit other forms of tobacco, would have wanted to seek treatment if they had to pay and were completely satisfied with the QTC (*p* < 0.05) (Table 3). The mean QTC visit frequency, counseling sessions, maximum abstinence from tobacco (months), quitting duration (months) and number of quit attempts were higher among those who had quit all forms of tobacco (*p* <0.05). Lastly, 68.5% were satisfied with the clinics and 82.5% were completely satisfied with staff behavior and 73.2% with counseling sessions (Table 4).

**Table 3:** Quitting all forms of tobacco by selected variables

		Quit all forms of tobacco	
		All forms	Not all
		*n* (%)	*n* (%)
Ever unemployed*	Yes	27 (45.8)	32 (54.2)
	No	83 (61.5)	52 (38.5)
Smoke cigarettes while ill*	Yes	60 (63.8)	34 (36.2)
	No	45 (49.5)	46 (50.5)
Reason not able to quit other product (s)*	Friends	8 (61.5)	5 (38.5)
	Alone	2 (33.3)	4 (66.7)
	Habituation	6 (20.0)	24 (80.0)
	Headache and tired	0 (0.0)	2 (100.0)
	Enjoy smoking	1 (7.7)	12 (92.3)
	Personal Problems	15 (39.5)	23 (60.5)
	Nervousness	4 (44.4)	5 (56.6)
Had received treatment if had to pay*	Yes	64 (64.0)	36 (36.0)
	No	46 (48.9)	48 (51.1)
Overall satisfaction with the clinics*	Not satisfied	3 (60.0)	2 (40.0)
	Slightly satisfied	1 (25.0)	3 (75.0)
	Satisfied	11 (39.3)	17 (60.7)
	Very satisfied	10 (41.7)	14 (58.3)
	Completely satisfied	85 (63.9)	48 (36.1

*P < 0.05

**Table 4:** Comparison of quitting all types of tobacco versus not quitting

	Quit tobacco	N	Mean	SD	Mean difference	95% CI		*P* value
						Lower	Upper	
Nicotine gum duration (days)	Yes	34	44.2	67.8	17.6	−26.40	61.72	0.423
	No	11	26.6	43.4		−18.26	53.58	
Nicotine patch duration (days)	Yes	29	30.4	27.7	4.3	−17.52	26.28	0.688
	No	13	26.0	41.5		−22.24	30.99	
Champix treatment duration (days)	Yes	110	97.2	13.3	2.5	−2.14	7.25	0.284
	No	84	94.6	19.8		−2.40	7.51	
Traditional herbal medicine duration (days)	Yes	110	99.0	0.0	2.3	−0.50	5.21	0.105
	No	84	96.6	15.2		−0.94	5.65	
Clinic visit frequency	Yes	81	3.3	2.2	1	0.35	1.82	0.004
	No	56	2.2	2.1		0.35	1.82	
Counseling/advice sessions	Yes	104	3.3	2.1	1.4	0.89	1.98	<0.001
	No	79	1.9	1.5		0.91	1.96	
Maximum abstinence from tobacco (months)	Yes	105	18.2	30.4	9.3	−1.6	20.28	0.095
	No	37	8.8	24.4		−0.63	19.26	
Quitting duration (months)	Yes	77	21.5	25.6	−17.1	−33.36	−0.93	0.038
	No	18	38.4	48.6		−1257.41	228.63	
Number of quit attempts	Yes	106	6.2	13.8	2.9	−0.11	6.09	0.059
	No	78	3.2	2.2		0.29	5.68	

## Discussion

The tobacco-quit rate of all forms of tobacco (56.5%) by QTC attendees is encouraging, indicating that the clinics contribute to the tobacco control efforts in the country and may decrease the health burden of tobacco smoking. It is also worth noting that almost two thirds of the attendees had no relapse after quitting.

Study participants smoked for a longer (19.8+ 12.8 years) time than that of *shisha* smokers (8.2 ± 6.1 years), which might also have contributed to the higher quit rates among the latter group. However, the fact that there were no statistically significant differences between those who completely quit all types of tobacco, cigarettes and *shisha* versus those who did not by smoking duration, frequency and quit attempts indicates that other factors might play a role in smoking cessation. The higher quit rates among *shisha* (63.8%) compared to cigarette smokers (55.1%) might suggest that quitting is easier among the former group. Studies on *shisha* intervention are scarce; however a recent review reported that interventions might help waterpipe smokers successfully quit smoking [11]. A study on waterpipe smokers in coffee shops in Bahrain reported that 82.0% expressed their ability to quit anytime, but only 40.0% wished to do so [12].

Cigarette smoking was the commonest form of tobacco smoked (96.9%). The majority smoked cigarettes alone (72.2%) without smoking other types of tobacco. The lower prevalence of *shisha* smokers attending the QTC can be partly attributed to the fact that *shisha* smoking may not be considered as addictive and harmful as cigarettes by the Bahraini community [13]; which warrants further efforts in educating the public about its harmful effects. Age started cigarette smoking (15.9 ± 4.3 years) was lower than that of *shisha* (19.7 ± 7.4 years). It was also slightly lower than that of cigarette smoking among the general male population (18.4 ± 5.2). Further, the average number of cigarettes smoked daily (27 + 16.9) was higher than that of the general adult population (19.35 ± 12.5) [14]. Unfortunately, there are no comparative data for *shisha* smoking with the general population.

The majority of the smokers knew about the QTC from their friends. This finding emphasizes the role of friends and peers not only in smoking uptake but also in smoking cessation. Further, the fact that wives asked their husbands to attend the clinic implies that they were worried about the health of their spouses and other members of the family being exposed to second hand smoke. It is disappointing that only 7.3% knew about the clinics from their physicians. This is not surprising as physicians in in the region have low perception of their role in helping smokers quit [15, 16].

Only 55.0% of the participants reported that their physicians asked them about smoking habits. Desire for quitting was related to physician’s advice, family’s attitude to *shisha* smoking, not considering own self being addicted and being a non-Bahraini [12]. This finding supports the important role of physicians in smoking cessation in Bahrain. It has been recommended in the smoking cessation guidelines for health professionals that they should advise smokers among their patients to quit and refer them to smoking cessation services if needed [17]. Although 33.0% of the participants reported that they received support from their physicians in quitting, it is disappointing that only 7.3% knew about the QTC from their physicians, in our study, although their advice on quitting tobacco smoking increases quit rates [18]. In Jordan, 19.9% had received advice from their physicians to stop smoking and only 2.4% had utilized the quitting cessation services [19].

The fact that 93.3% required NRT implies that the clinic attendees were those who could not successfully quit tobacco smoking through counseling alone, because of tobacco dependence. This also underscores the scale of economic burden on controlling tobacco smoking by the MOH in Bahrain and calls upon more efforts to be focused towards prevention of initiation of tobacco smoking.

Our study showed that clients who were able to quit all forms of tobacco had made one additional visit to the QTC (*p* = 0.004) and 1.4 more counseling sessions (*p* = <0.0001) than those who did not quit. We also found that quitters had abstained from smoking for 9.3 months more than the non-quitters (*p* = 0.038). The lower satisfaction rates related to clinic days and opening hours warrants attention. But the higher satisfaction rates with the staff and the counseling is rewarding.

Among the unemployed, there was a statistically significant (*p* < 0.05) relationship of successful quitting for those having less number of years of unemployment compared to their counterparts who had been unemployed for longer periods. This is probably due to the fact that the latter could be more stressed and could resort to tobacco dependence as a relief.

Over three quarters of the cigarette smokers smoked their first cigarette within the first half hour after waking up, which reflects that they were addicted to cigarette smoking. We found that cigarette smokers had a very high tobacco dependence that they would even smoke during illness (*p* < 0.05) and that most of them would not give up a morning cigarette which, along with the fact that they smoked an average of 27 cigarettes per day for the past 20 years. However their desire to quit tobacco was reflected by the fact that successful quitters reported that they could buy NRT even if they had to pay for it (*p* = 0.05). We did not find any significant difference in the successfully quitting with respect to the type and method of NRT used; however, as most quitters used a combination of a nicotine patch and gum we assume that it was the best method in Bahrain to practice and was found to be highly acceptable and convenient by the quitters.

### Strengths and limitations

The strength of our study was that it was the first of its kind in assessing a national tobacco intervention from a GCC country. Having QTC management as part of the study team would hopefully improve the quality of QTC services and healthcare policy makers in Bahrain would consider empowering these clinics and increasing their geographical distribution. The main study limitation was the low response rate. Several patients who attended the clinics early 2013 had changed their contact numbers and could not be traced. Others were reluctant to be interviewed. Since our sample was drawn from the patients who came to the health centers for tobacco cessation treatment, the quit rates may not reflect those of the general male population in Bahrain.

## Conclusions

The fact that more than half of the QTC attendees were able to quit tobacco emphasize the importance of these clinics in tobacco control in Bahrain. Better dissemination of information about the clinics by the healthcare providers is of paramount importance. Public awareness of the QTC clinics through educational establishments, schools, colleges, universities and media is of importance. The MOH may consider increasing QTC accessibility by increasing working hours and open days for the existing QTC.

## Abbreviations

BHD: Bahraini Dinar
EMR: Eastern Mediterranean Region
GCC: Gulf Cooperation Council
MOH: Ministry of Health
NCDs: Non-communicable diseases
NRT: Nicotine replacement therapy
PHC: Primary healthcare
QTC: Quit tobacco clinics
